# Head and neck melanoma (excluding ocular melanoma): United Kingdom National Multidisciplinary Guidelines

**DOI:** 10.1017/S0022215116000852

**Published:** 2016-05

**Authors:** O A Ahmed, C Kelly

**Affiliations:** 1 Department of Plastic and Reconstructive Surgery, The Newcastle upon Tyne Hospitals NHS Trust, Newcastle upon Tyne, UK; 2 Northern Centre for Cancer Care and Newcastle University, Freeman Hospital, Newcastle upon Tyne, UK

## Abstract

This is the official guideline endorsed by the specialty associations involved in the care of head and neck cancer patients in the United Kingdom. This paper provides consensus recommendations on the management of melanomas arising in the skin and mucosa of the head and neck region on the basis of current evidence.

**Recommendations:**

• At-risk individuals should be warned about the correlation between ultraviolet radiation (UVR) exposure and skin cancer, and should be given advice on UVR protection. (R)

• Dermatoscopy can aid in the diagnosis of cutaneous melanoma. (R)

• Histological examination after biopsy is essential to confirm the diagnosis and the tumour thickness. (G)

• Excisional biopsy is method of choice. (G)

• Staging investigations can be performed for both regional and distant disease. (R)

• Scanning (computed tomography (CT) and/or magnetic resonance imaging) is recommended for patients with high-risk melanoma. (G)

• Patients with signs or symptoms of disease relapse should be investigated by imaging. (R)

• Imaging of the brain should be performed in patients who have stage IV disease. (G)

• Patients with melanoma of unknown primary should be thoroughly examined and investigated for a potential primary source. (R)

• Primary cutaneous invasive melanoma should be excised with a surgical margin of at least 1 cm. (G)

• The maximum recommended excision margin is 3 cm. (R)

• The actual margin of excision depends upon the depth of the melanoma and its anatomical site. (G)

• Ultrasound-guided fine needle aspiration (FNA) or core biopsy of suspected lymphadenopathy is more accurate than ‘blind’ biopsy. (R)

• Open biopsy should only be performed if FNA or core biopsy is inadequate or equivocal. (R)

• Prior to lymph node dissection, staging by CT scan should be carried out. (R)

• If parotid disease is present without neck involvement, both parotidectomy and neck dissection should ideally be performed. (R)

• There is no role for elective lymph node dissection. (R)

• Sentinel lymph node biopsy (SLNB) can be considered in stage IB and above by specialist skin cancer multidisciplinary teams. (G)

• Patients should be made aware that SLNB is a staging procedure, and should understand that it has, as yet, no proven therapeutic value. (R)

• All patients with cutaneous melanoma should have their original tumour checked for BRAF gene status, and their subsequent targeted biological therapy based on this. (R)

• Patients who develop brain metastases should be considered for stereotactic radio-surgery. (R)

## Cutaneous melanoma of the head and neck

### Introduction

Cutaneous melanoma, also known as cutaneous malignant melanoma, is a malignant tumour of neural crest-derived cutaneous melanocytes. The incidence of melanoma has been increasing rapidly for the last few decades in most parts of the world. It is the fifth commonest cancer in the UK, with a male:female ratio of 10:11. The number of melanoma cases doubled in this country over the three decades following 1970. Over that same period the prognosis dramatically improved. This improvement is mostly attributable to a higher proportion of thinner tumours as a result of earlier diagnosis, and reflects the considerable effort expended in raising public and professional awareness over that period. Although melanoma is the major cause of skin cancer mortality, it is usually curable if treated at an early stage. Melanoma in its advanced stages remains largely resistant to currently available treatments, although in the last five years, new targeted biological agents and immunotherapies have offered the potential for improved survival.

### Aetiology and risk factors

Melanomas can arise in pre-existing naevi, or *de novo* in normal skin. Like most tumours, the aetiology of melanoma is complex and not fully understood. It is, however, thought to be caused by ultraviolet radiation (UVR) in susceptible individuals. It is estimated that around 86 per cent of melanomas in the UK in 2010 were linked to exposure to UVR from the sun and sun-beds.^1^ Fair-skinned individuals who burn easily in the sun, have fair or red hair, and have a tendency to freckle are about three times more likely to develop melanoma. A number of case–control studies conclude that intense burning sun exposure of unacclimatised white skin is a major risk factor for cutaneous melanoma. Migration studies show that exposure to intense UVR at a young age may be particularly important. This is in contradistinction to squamous cell and basal cell carcinomas, which are associated with chronic, long-term sun exposure. Patients with xeroderma pigmentosum have a significantly higher risk of all types of skin cancer, including melanoma, as a result of inability to repair the DNA damage induced by UVR.

**Recommendation**

• At-risk individuals should be warned about the correlation between UVR exposure and skin cancer, and should be given advice on UVR protection (R)

While it is understood that melanoma is related to UVR exposure, it is not clear why the body site distribution of melanoma is different to other sun-related cancers such as cutaneous squamous cell carcinoma. This suggests that the pattern of UVR exposure is important, with sites that are intermittently exposed being more at risk than continually exposed sites. The gaps in our knowledge of the aetiology have recently been critically evaluated.

Other risk factors include a large number of banal naevi, a tendency to freckle, and more atypical or dysplastic naevi.^2^ About 2 per cent of melanoma patients have a positive family history in one or more first degree relatives. The major melanoma susceptibility gene identified to date is CDKN2A gene. Mutations in this gene are found in 10–30 per cent of melanoma patients with a positive family history. Melanoma is more prevalent in those of high socio-economic status, but the converse applies to mortality.

### Clinical presentation

Cutaneous melanoma is divided into subtypes on the basis of clinical features and pathology.

#### Superficial spreading melanoma (SSM)

This is the most frequently encountered type of melanoma; characteristically an asymmetrical pigmented lesion with irregular borders, irregular pigmentation and sometimes an irregular outline. Patients may have noted growth, a change in sensation and/or colour, crusting, bleeding or inflammation of the lesion. The duration of the symptoms varies from a few months to several years.

#### Nodular melanoma (NM)

The second most common type of melanoma is NM. This usually has a shorter presentation and a greater tendency to bleed and/or ulcerate.

#### Lentigo maligna melanoma (LMM)

The next in frequency is the type that occurs most often in sun-damaged skin on the head and neck of older patients. This is the only variety that has a clearly recognised and often lengthy pre-invasive (*in situ*) lesion termed lentigo maligna (LM) before progressing in some instances to an invasive melanoma (LMM).

#### Acral lentiginous melanoma (ALM)

The least common type of melanoma in the UK is the ALM. This occurs on sites, including the palms, soles and beneath the nails. It is the most common melanoma found in African and Asian populations.

#### Desmoplastic neurotropic melanoma

This type is associated with higher local recurrence than other forms of melanoma. This is thought to be a consequence of its propensity for peri-neural spread. Desmoplastic neurotropic melanoma is predominantly found in the head and neck.

## Assessment and staging

Suspicious pigmented lesions are best examined in a good light with or without magnification and should be assessed using the seven-point checklist^3^ (Table I) or ABCDE systems (Table II). The presence of any major feature in the seven-point checklist, or any of the features in the ABCDE system, is an indication for referral. The presence of minor features should increase suspicion. Some melanomas will have no major features.

**Table I:** Seven point checklist for pigmented skin lesions

Major features	Minor features
Change in size of lesion	Inflammation
Irregular pigmentation	Itch/altered sensation
Irregular border	Lesion larger than others
	Oozing/crusting of lesion

**Table II:** The ABCDE checklist for pigmented skin lesions

**A**	Geometrical **A**symmetry in two axes
**B**	Irregular **B**order
**C**	At least two different **C**olours in lesion
**D**	Maximum **D**iameter >6 mm
**E**	**E**levation of lesion

Clinical diagnosis of melanoma can be difficult and the accuracy of diagnosis varies according to a clinician's level of experience, with reports of variation in sensitivity from 50 to 86 per cent. High magnification dermatoscopy is more sensitive than non-dermatoscopic diagnosis, when used by those trained and experienced in the technique.^4^ Hand-held (lower magnification) dermatoscopy improves diagnostic accuracy in those trained to be ‘expert’, but it may decrease diagnostic sensitivity of ‘non-expert’ or untrained dermatologists.

## Diagnostic biopsy

The thickness of cutaneous melanoma greatly influences both its treatment and its prognosis. It is essential, therefore, to obtain a full-thickness biopsy of suspected lesions. Excisional biopsy is the preferred technique, and is aimed at excising the lesion with a 2–5 mm peripheral margin, including a cut-off of subdermal fat. This allows accurate assessment of the tumour thickness and depth of penetration, without transgressing tumour boundaries. Excisional biopsy may not be practical when the lesion is large or located near structures such as an eyelid or lip. Punch biopsy is an alternative where excision biopsy could lead to significant disfigurement. A punch biopsy is usually performed with a 2–4 mm biopsy punch at the thickest or highest part of the lesion. Incisional biopsy is not usually recommended, but the indications are the same as those for punch biopsy. Again, it should be performed at the thickest or highest part of the lesion and must reach the full depth of the lesion

**Recommendations**

• Dermatoscopy can aid in the diagnosis of cutaneous melanoma (R)
• Histological examination after biopsy is essential to confirm the diagnosis and the tumour thickness (G)
• Excisional biopsy is method of choice (G)

## Staging

The latest revisions to the staging of cutaneous melanoma were published in the 7th Edition of the American Joint Committee on Cancer (AJCC) in 2009 (Table III).^5^ Of note, primary tumour mitotic rate (mitoses/mm^2^) is now considered an important independent prognostic indicator, with an inverse correlation between mitotic rate and survival. The mitotic rate replaces Clark's level of invasion as a primary criterion for separating T1 tumours into T1a and T1b.

**Table III:** TNM staging system for cutaneous melanoma

T classification	Thickness	Ulceration status/mitoses
Tis	N/A	N/A
T1	≤1.0 mm	a: w/o ulceration and mitoses <1/mm^2^
		b: with ulceration or mitoses ≥1/mm^2^
T2	1.01–2.0 mm	a: w/o ulceration
		b: with ulceration
T3	2.01–4.0 mm	a: w/o ulceration
		b: with ulceration
T4	>4.0 mm	a: w/o ulceration
		b: with ulceration
N classification	No of metastatic nodes	Nodal metastatic mass
N0	0 nodes	N/A
N1	One node	a: micrometastasis*
		b: macrometastasis†
N2	Two to three nodes	a: micrometastasis*
		b: macrometastasis†
		c: in-transit met(s)/satellite(s) without metastatic nodes
N3	Four or more metastatic nodes, or matted nodes, or in-transit met(s)/satellite(s) with metastatic node(s)	
M classification	Site	Serum lactate dehydrogenase (LDH)
M0	0 sites	N/A
M1a	Distant skin, subcutaneous, or nodal mets	Normal
M1b	Lung metastases	Normal
M1c	All other visceral metastases	Normal
	Any distant metastases	Elevated

* Micrometastases are diagnosed after sentinel lymph node biopsy and completion lymphadenectomy (if performed)

† Macrometastases are defined as clinically detectable nodal metastases confirmed by therapeutic lymphadenectomy or when nodal metastasis exhibits gross extracapsular extension

## Anatomical staging

**Table IV:** Clinical and pathological staging for cutaneous melanomas

Clinical staging*				Pathological staging†			
0	Tis	N0	M0	0	Tis	N0	M0
IA	T1a	N0	M0	IA	T1a	N0	M0
IB	T1b	N0	M0	IB	T1b	N0	M0
	T2a	N0	M0		T2a	N0	M0
IIA	T2b	N0	M0	IIA	T2b	N0	M0
	T3a	N0	M0		T3a	N0	M0
IIB	T3b	N0	M0	IIB	T3b	N0	M0
	T4a	N0	M0		T4a	N0	M0
IIC	T4b	N0	M0	IIC	T4b	N0	M0
III	Any T	N > N0	M0	IIIA	T1–4a	N1a	M0
					T1–4a	N2a	M0
				IIIB	T1–4b	N1a	M0
					T1–4b	N2a	M0
					T1–4b	N1b	M0
					T1–4b	N2b	M0
					T1–4b	N2c	M0
				IIIC	T1–4b	N1b	M0
					T1–4b	N2b	M0
					T1–4b	N2c	M0
					Any T	N3	M0
IV	Any T	Any N	M1	IV	Any T	Any N	M1

* Clinical staging includes microstaging of the primary melanoma and clinical and/or radiologic evaluation for metastases. By convention, it should be used after complete excision of the primary melanoma with clinical assessment for regional and distant metastases

† Pathological staging includes microstaging of the primary melanoma and pathological information about the regional lymph nodes after partial or complete lymphadenectomy. Pathological stage 0 or IA patients are the exception; they do not require pathological evaluation of their lymph nodes

## Imaging considerations

Staging investigations for regional lymph node metastases are often performed, and may comprise computed tomography (CT), magnetic resonance imaging (MRI), and/or ultrasound, depending upon local protocols. The use of scans to detect distant metastasis is indicated in patients with high-risk melanoma (stages IIC, IIIB, IIIC and stage IIIA with a macroscopic sentinel lymph node), and in patients with new symptoms, anaemia, elevated lactate dehydrogenase or a chest X-ray abnormality. Computed tomography scanning is used for the evaluation of potential metastatic sites in the lungs, bones, liver and lymph nodes. Imaging of the brain is recommended in patients with stage IV, but is optional in stage III disease. Positron emission tomography (PET)-CT is more accurate than CT or MRI alone in the diagnosis of metastases. It should complement conventional CT and MRI imaging in patients who have distant metastases and where surgical resection is being considered.

**Recommendations**

• Staging investigations can be performed for both regional and distant disease (R)
• Scanning (CT and/or MRI) is recommended for patients with high-risk melanoma (G)
• Patients with signs or symptoms of disease relapse should be investigated by imaging (R)
• Imaging of the brain should be performed in patients who have stage IV disease (G)

## Unknown primary

The patient presenting with regional or visceral metastatic melanoma of unknown primary (MUP) origin should be seen by a dermatologist for a skin examination, an ophthalmologist for examination of the eye, and a head and neck surgeon for visualisation of the upper aerodigestive tract. Staging investigations should be carried out, including PET-CT to detect occult metastases. In 10–20 per cent of patients with regional or visceral melanoma metastases, the primary melanoma is never found. Such patients should be treated as if they have regional or visceral metastases from a known primary melanoma.^6^ It has been suggested that the most likely explanation for MUP is immune-induced regression of the primary tumour, and this may be the reason for the slightly better outcomes in such patients.

**Recommendation**

• Patients with a melanoma of unknown primary origin should be thoroughly examined and investigated for a potential primary source (R)

## Management

### Surgery for primary disease

#### Wide local excision

This remains the most effective treatment for primary cutaneous melanoma.^2^^,^^7^ The optimal width of excision margins has been contentious.^8^^,^^9^ Current treatment guidelines are based on a relatively small number of prospective randomised trials.^10^^–^^12^ The current recommended excision margins for cutaneous melanoma in the UK are as follows:

• In situ melanoma (LM): 5 mm peripheral margins
• Lesions <1 mm thick: 1 cm excision margins
• Lesions 1–2 mm thick: 1–2 cm excision margins
• Lesions 2.1–4 mm thick: 2–3 cm margins (2 cm preferred)
• Lesions thicker than 4 mm: 2–3 cm margins.

It should be stressed that these recommendations are for cutaneous melanomas in all body sites; in the head and neck region, anatomical restrictions and cosmetic considerations may preclude even a 1 cm margin. In these circumstances, however, the width of excision should remain uniform. For example, if a clear margin of only 8 mm is possible near to an eyelid or an ear, the rest of the peripheral surgical margins should also be 8 mm.

**Recommendations**

• Primary cutaneous invasive melanoma should be excised with a surgical margin of at least 1 cm (G)
• The maximum recommended excision margin is 3 cm (R)
• The actual margin of excision depends upon the depth of the melanoma and its anatomical site (G)

#### Mohs micrographic surgery (MMS)

Mohs micrographic surgery may have a role in the primary treatment of cutaneous melanoma of the head and neck, especially that of the face.^13^ There is growing evidence of the efficacy of MMS in comparison to traditional surgery but the majority of reports compare MMS with historical controls. Further study, in the form of prospective comparative trials, is required before firm recommendations can be made regarding the use of MMS.

#### Reconstruction

When possible, the surgical defect after wide local excision should be closed primarily. If primary closure is not possible, reconstruction by local flaps or skin grafts will be required. Local flaps are the preferred option when the surgical defect is on the face, because of a superior aesthetic outcome. Rarely, distant flaps will be required for complex or very large surgical defects. If there is any doubt as to the adequacy of surgical clearance, definitive reconstruction should be delayed pending histological confirmation.

### Surgery for regional disease

The regional lymph node basin in head and neck cutaneous melanoma comprises the nodes found in the parotid gland (superficial portion), neck levels I–V, the occipital nodes, mastoid nodes and pre-auricular nodes. There may be clinically apparent lymphadenopathy, representing metastatic melanoma, or occult metastases in the head and neck nodes.

#### Clinical lymphadenopathy

When patients present with a neck mass or a radiologically identified suspicious node(s) a tissue diagnosis should be obtained. The preferred stepwise diagnostic algorithm to follow is: (1) palpable lymph node in the neck or radiologically identified suspicious node; (2) ultrasound-guided or clinically-guided FNA; (3) ultrasound-guided core biopsy; (4) open biopsy.

Fine needle aspiration is more accurate when performed with ultrasound guidance, and this should be subsequently performed if a clinically guided FNA is non-diagnostic. If an open biopsy is performed, the incision should be placed in a manner which permits subsequent excision of the biopsy tract if a neck dissection is necessary. If metastatic melanoma is confirmed, lymphadenectomy of the involved nodal basin should be performed.

The extent of lymphadenectomy performed for melanoma is determined by the location of the primary, the location of the neck disease, and the general fitness of the patient. If parotid lymphadenopathy is present, a neck dissection should also be performed as a high proportion of patients with parotid lymph node involvement will have occult cervical metastases. If neck disease is present without parotid involvement then the location of the primary should be considered. If the draining basin of that primary site is likely to pass through the parotid gland, a concomitant superficial parotidectomy should be considered. It is reasonable to perform a selective neck dissection for some melanoma sites that have metastasised to the neck when there is low volume, mobile lymphadenopathy. For example, omitting excision of level IA and IB neck nodes for a well-lateralised occipital melanoma would be accepted management.

**Recommendations**

• Ultrasound-guided FNA or core biopsy of suspected lymphadenopathy is more accurate than ‘blind’ biopsy (R)
• Open biopsy should only be performed if FNA or core biopsy is inadequate or equivocal (R)
• Prior to lymph node dissection, staging by CT scan should be carried out (R)
• If parotid disease is present without neck involvement, both parotidectomy and neck dissection should ideally be performed (R)
• If neck disease is present without parotid involvement, parotidectomy should be considered if the lymphatic drainage of the primary site is likely to have passed through the parotid gland (R)

#### Occult lymph nodal disease

The most accurate means of staging the regional lymph nodes in head and neck melanoma is by sentinel lymph node biopsy (SLNB). This staging tool has a learning curve and involves the administration of a radiocolloid into the site of the excision biopsy. Pre-operative lymphoscintigraphy identifies the approximate location of the sentinel nodes and the intra-operative use of blue dye and a gamma probe aids in location of the sentinel node(s). The removed sentinel nodes are histologically examined with multiple sections and immunohistochemical stains for the presence of occult metastases. Sentinel lymph node biopsy identification of regional lymph node metastasis should be followed by lymphadenectomy of the at-risk nodal basin.

Whether or not SLNB is performed for staging the regional lymph nodes is a matter for local policy. Sentinel lymph node biopsy provides highly accurate staging information but there is controversy as to whether or not it improves disease-specific survival. The long-term results of the Multicentre Selective Lymphadenectomy Trial-I (MSLT-I) indicate that SLNB is associated with improved disease-free survival for patients with intermediate thickness (1.2–3.5 mm) and thick (≥3.5 mm) melanomas,^14^ but this has been questioned in a recent editorial in the *British Medical Journal*.^15^ Furthermore, there is the question of biological false-positivity.^16^ Some clinical trials require information on disease stage and an SLNB can provide this information. Sentinel lymph node biopsy has replaced elective lymph node dissection in melanoma and there are few indications to perform the latter.

The Options Grid Collaborative, based at Dartmouth University, is an organisation which attempts to improve shared decision-making between healthcare professionals and patients, their carers and families. They produced, in collaboration with the National Institute for Health and Care Excellence, three tools to try and help patients with practical decision-making in managing melanoma. These can be found at http://optiongrid.org

**Recommendations**

• There is no role for elective lymph node dissection (R)
• Sentinel lymph node biopsy can be considered in stage IB and above by specialist skin cancer multidisciplinary teams (G)
• Patients should be made aware that sentinel lymph node biopsy is a staging procedure, and should understand that it has, as yet, no proven therapeutic value (R)

##### Metastatic disease

Distant melanoma metastases occur preferentially and earliest in intra-abdominal organs, liver, lung, brain and bone. Whilst these are the commonest sites, metastases to almost every organ and tissue have been reported.

Distant metastases can be divided into two groups: metastases already established at presentation of the primary (stage IV disease) and metastases that subsequently become apparent. Metastases at presentation carry the worst prognosis, while for delayed metastases the prognosis improves in direct proportion to the time taken for the metastasis to develop. In practice the questions to be addressed are what, if any, improvement in survival time may be gained from treatment of metastatic disease and what symptomatic improvement will occur?

Treating metastases in patients with distant metastases confirmed at presentation (stage 4 disease) has proved very disappointing. Survival rates in such individuals have not improved over the last two decades.

Resection of late-appearing metastases to non-liver intra-abdominal organs or gastro-intestinal mucosa yields the best improvement with a disease-free survival in the region of 23 months compared with a median survival of only 12 months if untreated.^17^ Patients undergoing surgical resection of late-appearing metastatic melanoma to the liver also have improved disease-free survival compared with untreated patients.^18^^,^^19^

Surgical resection of pulmonary metastases and solitary brain metastases from melanoma may yield a survival advantage of a few months more than any other method of dealing with these lesions. Stereotactic radiosurgery (SRS) for brain metastases also offers some patients extended survival. Early treatment of spinal cord secondaries can preserve mobility. Bone metastases are associated with short survival irrespective of treatment.

Biological markers have been studied extensively in metastatic melanoma with regard to prognosis and as a guide to resectability of metastases. Of these, lactate dehydrogenase (LDH) and the c-kit mutation may be helpful. A high serum LDH level suggests a large disease burden and a poor result from treatment of metastases.

Aggressive surgical treatment of distant metastases from melanoma at any site must be carried out on highly selected patients and, even then, it is best regarded as a palliative procedure, usually improving survival by only a matter of months. Nevertheless, quite long remissions may be obtained in fit patients with apparently solitary, or oligometastatic, disease.

#### Non-surgical treatment

##### Primary tumour

There is no established role for primary radiotherapy (RT) (instead of surgery) in the management of early stage (stages Ia, b and IIa, b, c) malignant melanoma, other than in elderly patients with extensive facial LMM. It is unlikely that this situation will change in the foreseeable future. Similarly, chemotherapy, biological agents and immunotherapy have no proven place in the management of early stage melanoma.^20^

##### Regional disease

In patients with stage III (nodal) or IV (M1) disease, the prognosis is significantly worse. Surgery remains the key initial treatment with the goal of securing local control, even in the setting of metastatic disease. There is no established role for RT in the management of patients with micrometastatic nodal disease (N1a, N2a). These patients are treated with surgery alone (±entry into studies of adjuvant systemic therapies). Recent adjuvant trials in malignant melanoma have included those testing immunotherapies (interferon, interleukin-2, peptide gp100:209–217(210 M), Canvaxin^TM^) and anti-angiogenic agents, such as bevacizumab (Avastin).^20^ For patients with macrometastatic nodal disease (N1b, N2b), there is no consensus that RT is beneficial following surgery, but for patients with cervical lymph node disease it is frequently used. There is no currently defined role for chemoradiation in this setting. The findings of the Phase III TROG 02.01 trial suggest that entry to an adjuvant systemic therapy trial may be a preferable alternative to adjuvant RT.^21^

##### Distant metastases

Patients with established metastatic melanoma are treated with palliative intent and should be referred to specialist melanoma units.

The chemotherapy management options for metastatic melanoma have greatly expanded in the last five years with the introduction of biologically targeted agents.^22^ About 50 per cent of melanomas show a mutation in the BRAF gene, with valine substituted for glutamate at codon 600, and this mutation is known as V600E or V600K. If this mutation is present then patients will have a 60–70 per cent chance of responding to a BRAF inhibitor drug such as vemurafenib^23^ or dabrafenib. Those patients who develop the most slowly but continued response to these BRAF inhibitors appear to achieve a more sustained response when compared with those patients who develop a very rapid tumour clearance. One potential sideeffect of these drugs, which must be monitored, is the development of squamous cell carcinoma of the skin.

The second major advance in the management of metastatic melanoma was the introduction of immunotherapy. Ipilimumab is a monoclonal antibody which targets cytotoxic T lymphocyte-associated protein 4 (CTLA-4) which is a protein receptor which can be made to switch off cytotoxic T lymphocytes (CTLs) by melanoma cells. Ipilimumab removes this brake on the immune response and allows the CTLs to recognise and destroy melanoma cells. This agent's efficacy does not depend on the patient's BRAF status. Although the response rate is only 15–20 per cent, in those patients who do show a response this can be sustained for some considerable time. There are hopes that some patients may have even been cured but follow-up has generally not been long enough to establish this.

There is much interest in metastatic melanoma at present, with numerous trials underway, especially in combining targeted therapies where by blocking two different steps in the same pathway a much greater melanoma cell kill may result. Another drug which blocks a specific pathway target is trametinib which is a MEK inhibitor. When combined with dabrafenib, there is both a progression free survival benefit and an overall survival benefit. Another MEK inhibitor cobimetinib, shows benefit when given with vemurafenib, when compared with giving vemurafenib alone.

Nivolamab is also a novel agent. It is a programmed death 1 (PD-1) checkpoint inhibitor which also shows complimentary benefit in metastatic melanoma when given together with ipilimumab, compared with each drug alone and this is now proposed as a standard of care in those patients who have wild-type BRAF, i.e. not showing a BRAF mutation. If this regimen does become standard of care it may not remain so for very long as the field is advancing so quickly.

For patients who become refractory to ipilimumab, and to the BRAF and MEK inhibitors there is a further new agent pembrolizumab, which targets the PD-1 receptor, and can extend progression-free survival.

Palliative RT is often used in metastatic disease (stages IV, M1a–c). Radiotherapy dose fractionation is non-standard in most of these treatments. Commonly used regimens include 8 Gy single fraction, 20 Gy in five fractions, 30 Gy in six fractions (alternate days), the latter fractionation being used most commonly for all brain RT for brain metastasis, and a host of local variations in different RT departments. There is no accepted role for the use of concomitant chemotherapy (although temozolomide has been tested with RT in cerebral metastases). There is emerging evidence that SRS can be beneficial in those patients who have a small number of brain metastases, usually less than three, where very focused high-dose RT can be given to the metastases, with very little dose to the surrounding brain.

**Recommendations**

• All patients with cutaneous melanoma should have their original tumour checked for BRAF status, and their subsequent targeted biological therapy based on this (R)
• Patients who develop brain metastases should be considered for stereotactic radiosurgery (R)

## Mucosal melanoma (upper aerodigestive tract)

### Introduction

Mucosal melanoma of the upper aerodigestive tract is a rare malignancy with a poor prognosis. Management recommendations are based upon retrospective case series, few of which exceed 100 patients. Mucosal melanoma accounts for less than 1 per cent of all melanomas, and less than 10 per cent of all head and neck melanomas. The median age of patient presentation is the sixth decade, but case reports span the age range.

The function of melanocytes in mucosa is uncertain. The most common sites of head and neck mucosal melanoma are the nasal and oral cavities. Pharyngeal, laryngeal and oesophageal melanomas are exceedingly rare. Melanocytes in the nasal cavity can be found in the respiratory epithelium, nasal glands, nasal septum, and the middle and inferior turbinates. In oral mucosa, melanocytes are located along the tips and peripheries of the rete pegs. Unlike cutaneous melanoma, no risk factors for the development of this disease have been identified, though cigarette smoke and other air pollutants may play a role. It is thought that a preceding atypical melanocytic hyperplasia occurs in a significant proportion.

### Clinical presentation

Sinonasal melanoma presents in the same way as other sinonasal malignancies and is primarily influenced by site of origin. Nasal obstruction, followed by discharge and bleeding, predominates. The commonest site is the anterior portion of the nasal septum. Oral mucosal melanoma most often presents as a painless mass, which may or may not be pigmented. Ulceration and bleeding are also common. The majority occur on the alveolar gingiva and palate.

### Assessment and staging

Endoscopic assessment and imaging, as appropriate to the primary site, is necessary, following which staging is performed (Table V).

**Table V:** TNM staging system for mucosal melanomas

I–Primary tumour			
TX	Primary tumour cannot be assessed			
T3	Epithelium and/or submucosa (mucosal disease)			
T4a	Deep soft tissue, cartilage, bone, overlying skin			
T4b	Brain, dura, skull base, lower cranial nerves (IX, X, XI, XII), masticator space, carotid artery, prevertebral space, mediastinal structures			
N–Regional lymph nodes			
NX	Regional lymph nodes cannot be assessed			
N0	No regional lymph node metastasis			
N1	Regional lymph node metastasis			
M–Distant metastasis			
M0	No distant metastasis			
M1	Distant metastasis			
Stage grouping:			
Stage III	T3	N0	M0
Stage IVA	T3	N1	M0
	T4a	N1	M0
Stage IVB	T4b	Any N	M0
Stage IVC	Any T	Any N	M1

Note: Mucosal melanomas are aggressive tumours, therefore T1 and T2 are omitted as are stages I and II

### Management

The prevailing opinion is that localised disease is best managed with primary surgery which aims to achieve clear surgical margins.^9^ Craniofacial resection for skull base extension from sinonasal melanoma is associated with poor survival and is seldom justified. Radical surgical excision involving severe functional deficits should not be performed in the context of established metastatic disease.

Reports indicate high rates of local recurrence (31–85 per cent), regional recurrence, and distant metastasis (25–50 per cent) as well as poor five-year survival rates (13–48 per cent), and a median survival of less than two years, for head and neck mucosal melanoma. The predominant mode of treatment failure is local recurrence, and this usually occurs within a year of initial treatment. It is frequently accompanied by the appearance of regional and distant metastases. Distant metastasis is associated with short survival time.

While the view of mucosal melanoma as a ‘radioresistant’ tumour has been challenged, the role of post-operative RT remains unclear. Its use has been reported to improve local control. Short-course, hypofractionated schedules (e.g. 30 Gy in six fractions over two weeks, 50 Gy in 20 fractions), to relatively small volumes, compared with other head and neck practice are frequently employed. Adjuvant chemotherapy and biological therapeutic strategies have been employed with encouraging response rates. For metastatic disease, unfortunately only a tiny percentage of mucosal melanomas show a BRAF mutation; therefore it is usually not appropriate to use BRAF inhibitors, so chemotherapy in the form of biological agents has to depend on immunotherapy with ipilimumab^24^ or the newer agents such as pembrolizumab, although to date there has been no specific study of the latter agent's efficacy specifically in mucosal melanoma.

### Key points

• Cutaneous melanoma is the fifth commonest cancer in the UK; the incidence of melanoma doubled in the three decades following 1970
• Despite widely used checklists, the clinical diagnosis of melanoma can be difficult and a biopsy is needed for diagnosis
• The thickness of cutaneous melanoma greatly influences both its treatment and its prognosis
• Staging includes microstaging of the primary melanoma and clinical/radiological evaluation for metastases
• Mucosal melanoma is a poor prognostic disease
• Wide local excision, with appropriate margins based on the thickness of the tumour, with or without lymph node dissection of the involved nodal basins, is the mainstay of treatment for primary cutaneous melanoma
• Excision of localised mucosal melanoma with clear margins is the mainstay of treatment, but radical excision with functional compromise has not shown oncological benefits. Advances in immunotherapy have revolutionised the management of distant metastases
